# Images of Eyes Enhance Investments in a Real-Life Public Good

**DOI:** 10.1371/journal.pone.0037397

**Published:** 2012-05-18

**Authors:** Damien Francey, Ralph Bergmüller

**Affiliations:** Department Eco-Ethology, University of Neuchâtel, Neuchâtel, Switzerland; Hungarian Academy of Sciences, Hungary

## Abstract

A key issue in cooperation research is to determine the conditions under which individuals invest in a public good. Here, we tested whether cues of being watched increase investments in an anonymous public good situation in real life. We examined whether individuals would invest more by removing experimentally placed garbage (paper and plastic bottles) from bus stop benches in Geneva in the presence of images of eyes compared to controls (images of flowers). We provided separate bins for each of both types of garbage to investigate whether individuals would deposit more items into the appropriate bin in the presence of eyes. The treatment had no effect on the likelihood that individuals present at the bus stop would remove garbage. However, those individuals that engaged in garbage clearing, and were thus likely affected by the treatment, invested more time to do so in the presence of eyes. Images of eyes had a direct effect on behaviour, rather than merely enhancing attention towards a symbolic sign requesting removal of garbage. These findings show that simple images of eyes can trigger reputational effects that significantly enhance on non-monetary investments in anonymous public goods under real life conditions. We discuss our results in the light of previous findings and suggest that human social behaviour may often be shaped by relatively simple and potentially unconscious mechanisms instead of very complex cognitive capacities.

## Introduction

A central issue in evolutionary biology is to understand cooperative behaviour among unrelated and unfamiliar individuals [1], [2], [3], [4], [5]. A main topic in this context is the question why individuals invest in a public good that everyone is free to exploit or overuse [6]. Without mechanisms to prevent exploitation, such public resources are predicted to collapse and end up in the so-called “tragedy of the commons” [7]. To understand under which conditions individuals choose to invest in a public good is not only of theoretical interest, but also of practical importance as some major problems humans face today, such as climate change, the financial crisis and over-exploitation of natural resources, ultimately result from a lack of cooperation in social dilemma situations [8], [9]. A social dilemma arises when all individuals enjoy the collective benefits of a public resource (such as an intact atmosphere) that can only be sustained when individuals refrain from over-using it, but economically rational actors do best by free riding. Similarly, individuals that do not contribute in producing or maintaining a public good (such as a clean environment) do best by profiting from, but not contributing to, the investments of others [10]. While a number of studies indicate that a significant fraction of individuals tend to start out cooperatively, the negative feedback resulting from defecting individuals typically causes a cooperation break down [11], [12]. However, the tragedy is not inevitable as we frequently do observe cooperation in social dilemma situations. Therefore, the question emerging is: which mechanisms contribute to maintain cooperation in such situations?

Research over the last three decades suggests that one important mechanism to explain cooperation in such situations is reputation or ‘indirect reciprocity’ [13], [14]. The underlying logic is that individuals with a good reputation receive more help than individuals with a worse reputation. Therefore, as helping others enhances reputation, individuals should behave more cooperatively when observed by others compared to when they are unobserved. Theoretical models have shown that cooperation can be maintained if individuals use information about the previous interaction of individuals and are more likely to cooperate with individuals that have helped others before. Individuals with a good reputation should be preferred as cooperation partners and therefore defecting individuals incur indirect costs resulting from a poor reputation [15]. This has recently been demonstrated empirically [16], [17]. A number of controlled lab experiments have also shown that individuals increase their cooperative investments when they know their behaviour is monitored [18], [19], [20]. Moreover, examples from changes of the behaviour and policy of large institutions also illustrate the power of reputational effects. For instance, since a few years leading financial institutions demand an environmental and social risk assessment before financing large scale industrial projects as part of their risk and reputation management strategy [21].

One approach to test whether individuals modify their behaviour in the presence of others is to investigate whether they respond to being watched. For instance, mutual eye gaze has been shown to increase contributions to a public good without any change in anonymity [22]. Recent studies found that individuals respond to more subtle cues, such as the presence of abstract eye-like spots on the background of the computer on which they complete a task [23], [24], [25], [26]. Even though relatively abstract cues elicited behavioural responses in some of the studies, most of them were conducted under somewhat artificial lab conditions, which may limit the applicability of the results to real world interactions. However, two studies also suggest that images of eyes enhance cooperation under real life settings [27], [28]. In one of these studies, images of pairs of eyes enhanced contributions to a honesty box used to collect money for drinks in the coffee room of a research group at the University of Newcastle compared to controls (images of flowers). Hence, cues of being watched appeared to enhance the exchange of money for an open uncontrolled resource (milk, which was a proxy for tea and coffee) due to reputational effects. Another more recent study showed that images of eyes decrease the likelihood that people would leave litter at their table in a University cafeteria [28].

In the present study, we aimed at going one step further in several aspects to investigate the effects of simple cues of being watched under realistic conditions. We asked whether individuals would invest in a public good (a clean environment) even without receiving any material good (such as money) in exchange. Furthermore, we investigated whether images of pairs of eyes would enhance investments (removal of foreign garbage) among unfamiliar individuals at public locations. We tested 14 different bus stops; hence, in contrast to many studies that only used students at the University, our sample comes closer to a random sample of the population. In order to obtain information about the precise investments of each individual we directly observed people's behaviour. Finally, we combined our behavioural data with a questionnaire to investigate whether images of eyes have a direct effect on cooperative behaviour, or whether the presence of eyes caused an increase in investment merely by enhancing attention towards a sign that requested cooperative behaviour (here: disposal of garbage).

If reputational effects cause an increase in investment in a public good, we predicted that cues of being watched (an image of eyes) enhance the likelihood and the amount of investment involved in removing garbage from a public location (a bus stop bench) compared to controls (an image with flowers). If images of eyes have a direct effect on behaviour that is not mediated by an increase in attention towards a sign requesting garbage removal, we predicted that the sign would not be noticed more often in the presence of eyes, compared to in the control.

## Methods

### (a) Experiment

**Figure 1:**
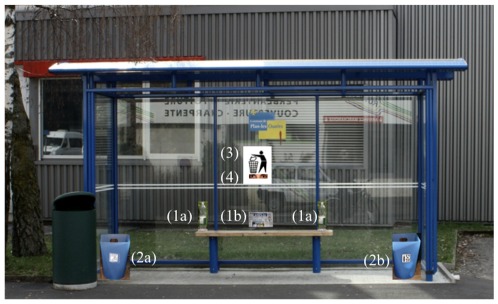
Experimental set up at bus stations. Three items of garbage (2 PVC bottles (1a) and a newspaper (1b)) were placed on the bus stop bench. Two experimental wastebaskets with signs, one for plastic (2a) and one for paper (2b) were placed, one at each side of the bus stop bench. Above the bus stop bench (in eye height, about 1.5 m) a symbolic sign (3) indicating to throw away garbage was attached. Below the sign either an image of eyes or flowers (4) was placed.

**Figure 2:**
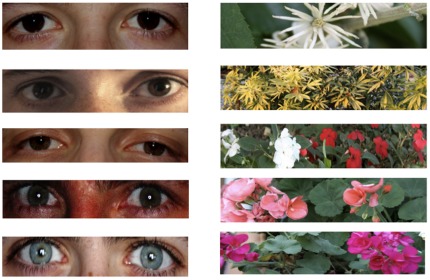
Treatments (images of eyes) and controls (images of flowers).

DF conducted the experiments at 14 different bus stops in Geneva (Switzerland). The tests were carried out at bus stops that were equipped in a similar way (with a bench and a bus stop shelter, Fig. 1). We selected bus stops according to the following criteria: (1) opposite bus stops allowed us to observe the subjects inconspicuously so they did not feel monitored by the observer, (2) the duration between successive buses allowed for observation and asking questions to the subject (at least 8 minutes between successive busses). Each bus stop was only used once (throughout one experimental day) in order to avoid to repeatedly testing the same persons. Before start of the experiment we placed two garbage bins with signs (one for plastic and one for paper garbage) at both sides of the bench so that the garbage could be separated according to garbage type. We placed three items of garbage on the bus stop bench (two empty plastic bottles (PET) of 1.5 l volume and a news paper) in such a way that the subjects needed to move away at least one item in order to sit down (Fig. 1). We attached a sign (Fig. 2) requesting that garbage should be thrown away above the bus stop bench (approximately at eye level, i.e. about 1.5 m high) so that each person in front or within the bus stop would be likely to notice it (Fig. 1). Below the sign we either attached one of five different photos of a pair of eyes, or one of five different images of flowers (size 12.0×2.8 cm) (Fig. 2).

Each test started when a bus left the bus stop, so there was a “clean” experimental situation without any people present. We observed the behaviour of the first subject approaching the bus stop for 2 minutes and recorded the following information: whether or not subject handled (handling refers in all cases to deposition of objects into the bins) garbage, duration of handling items (seconds), location of depositing the items (in which of the two bins the item was deposited), and whether subjects remained standing or sat down on the bench. Furthermore, we recorded the gender of the person. Two minutes after arrival of the subject, we approached the subject to conduct our questionnaire. Treatments (eyes/flowers) were exchanged about every 2 hours and the type of eye and flower was presented in random order.

### (b) Questionnaire

After the observations ended we approached the persons and asked the following questions: Did you notice the sign? Did you notice the eyes/flowers? Did you feel observed by me (the observer)? After the questionnaire, subjects were informed about the underlying reasons for the experiment and the questionnaire. All data were recorded and analysed anonymously.

### (c) Data analyses

We only included observations in our analyses in which the conditions concerning our question of interest were appropriate, i.e., subjects were alone at the bus stop for at least 2 minutes and subjects positioned themselves in front of the bus stop so they could see, and potentially be affected by the sign and the experimental garbage. We first analysed the overall effect of the treatment on whether or not individuals engaged in garbage clearing. For the detailed analyses of behavioural differences depending on the treatment we only analysed those cases in which people engaged in garbage removal. This is because individuals that did not clear garbage are neutral to the treatment in the sense that it is not possible to assess the effects of the treatment on their behaviour. In a first step, we tested whether individuals invested more time in handling garbage in the presence of images of eyes compared to the control. In a second step, we investigated whether the amount of time invested was positively correlated to the number of items handled, the number of items place into the correct bin or, the proportion of items placed into the correct bin (precision). Because of the low sample size of people that were willing to respond to our questionnaire, we included all available responses to the questionnaire in the analyses, including those of people that were excluded from the behavioural analyses because of methodological considerations (e.g., when the person was not alone during two minutes after arrival). As some individuals left with the bus before they had answered the full questionnaire we do not have the same amount of answers for each question (between 18–30 answers per question could be analysed). All results reported are two-tailed and were performed with SPSS version 16.0. P-values<0.05 are considered as statistically significant and p-values> = 0.05 but <0.1 are reported as non-significant trends.

### (d) Ethical note

For our study we did not obtain a formal ethical permit from the ethical board because we instead avoided all manipulations that could have raised ethical concerns. We adhered to the Ethical standards of the Suisse Society for Psychology (Schweizer Gesellschaft für Psychology) and to the checklist for ethical consideration of psychological studies (“Checkliste für die ethische Beurteilung von Psychologischen Forschungsvorhaben”, see www.ssp-sgp.ch/ethik.htm). According to this checklist, we avoided any treatments that might have negatively affected the psychological or social integrity of our study subjects in any way. In detail, we did not use any invasive methods in our experiment, but observed behaviour in the field using a small experimental manipulation. We presented images of eyes at bus stop benches to investigate whether persons would be more inclined to remove experimentally applied garbage into garbage bins. This small manipulation of the environment can safely be assumed not to be harmful to persons in any way. According to the Ethical standards, we did not apply aversive stimuli or induce negative emotions in any way. Also, our experiment did not have any negative impacts on the reputation of the tested persons as they remained fully anonymous. We did not collect any personalised data and did not film or tape the behaviours and responses of the test persons. We did not ask questions to persons that were underage or otherwise potentially inhibited in their ability to judge persons. Test subjects were fully free not to respond to our questions and were informed and asked for consent to use their anonymous responses for the purpose of our study after responding. Our questionnaire was designed so it did not contain any questions that asked for potentially emotionally disturbing personal experiences or political preferences.

## Results

### (1) Did images of eyes enhance investments in a public good?

About one third (28 of 93) of the subjects placed garbage into the bins. When testing all persons present at the bus stop, the treatment had no significant effect on the likelihood that individuals would handle (handling allways refers to depositing objects into the bins) garbage items (N_Flowers_no-handling_ = 32; N_Flowers_handling_ = 12; N_Eyes_no-handling_ = 33; N_Eyes_handling_ = 16; Chi^2^ = 0.32; p = 0.57). Individuals who sat down on a bus bench tended to be more likely to handle garbage items compared to individuals who remained standing (Chi^2^: N_Standing_no-handling_ = 32; N_Standing_handling_ = 8; N_Sitting_no-handling_ = 33; N_Sitting_handling_ = 20; Chi^2^ = 3.41; p = 0.065).

**Figure 3:**
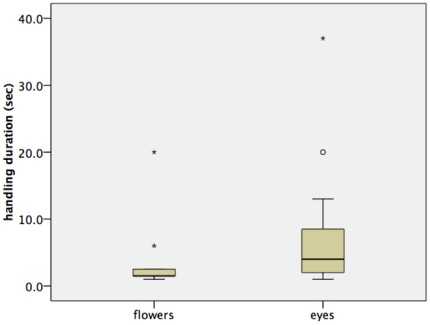
Handling duration of garbage was increased in the treatment (image of eyes) compared to the control (image of flowers).

Using only the data from people that engaged in handling garbage (and therefore were likely subject to the treatment), people invested about twice the time in handling garbage in the presence of eye images as opposed to flower images (Fig. 3; Mann-Whitney U test: N_Eyes_ = 16; N_Flowers_ = 12; Z = −2.17; p = 0.03). Half of these persons (14 out of 28) removed and deposited more than one garbage item into the bins, sometimes up to all three items.

The amount of time people invested in handling garbage items correlated positively with the number of items handled (Spearmen's rank correlation, N = 28, c = 0.67, p<0.001) and with the number of items people deposited into the correct bin (N = 28, c = 0.50, p = 0.007). However, the proportion of items deposited into the correct bin (i.e. precision) was not correlated with handling duration (N = 27, c = 0.08, p = 0.69). Significant results remain significant when adjusting the critical p-value for multiple testing (critical p = 0.017).

### (2) Did images of eyes have a direct effect on behaviour?

Using the data from the questionnaire we tested whether eyes could have caused the effect of higher investment in the treatment by raising the attention towards the sign that instructed subjects to throw away garbage. We analysed whether individuals noticed the sign more often when eyes were present compared to the control. Individuals noticed the sign more often when flowers were present compared to in the presence of eyes (N_Flowers_not noticed_ = 5; N_Flowers_noticed_ = 8; N_Eyes_not noticed_ = 13; N_Eyes_noticed_ = 4; Chi^2^ = 4.43; p = 0.042), suggesting that the effect of the eyes cannot be explained by increased attention towards the sign requesting garbage removal. There was no difference concerning how often people reported to have noticed the experimental eyes or flowers (N_Flowers_not noticed_ = 8; N_Flowers_noticed_ = 5; N_Eyes_not noticed_ = 12; N_Eyes_noticed_ = 5; Chi^2^ = 0.27; p = 0.60). None of 18 individuals responded to have felt observed by the experimenter in the opposite bus stop (N_Flowers_ = 8, N_Eyes_ = 10).

## Discussion

A central issue in the study of human cooperation is the question about which mechanisms promote investments into public goods. While theoretical and lab studies showed that reputation can enhance cooperation under certain conditions [14], [18], [19], [29], as yet only two experimental studies investigated whether reputational effects can enhance cooperation in realistic settings [27], [28]. In accordance with the previous studies, our results suggest that simple cues of being watched can enhance investments in a real life public good. Eye images resulted in a significant increase in the amount of time that people spent removing garbage as people spent about twice the time handling garbage in the treatment. While people invested in depositing the items into the appropriate bin, there was no evidence that treatment affected precision (proportion of items deposited into the correct bin). In accordance with a previous study [28] our results suggest that it was indeed the cues of eyes that caused this effect (not increased attention towards the sign requesting removal of garbage) as persons noticed the sign that requested people to deposit garbage more often in the presence of flowers compared to in the presence of images of eyes. Our study provides evidence in a real life situation in humans that subtle reputational effects such as simple cues of being watched can enhance investments in public goods among anonymous and unfamiliar individuals.

### (a) Why did individuals invest in public goods?

Reputation has been shown to foster cooperation also in public good games [19]. However, the idea that simple images of eyes could result in reputational effects on human behaviour seem surprising at first glance. This is because this implies that images of eyes can shape behaviour despite of the absence of real human observers. Cues of being watched appear to work by means of evolved psychological mechanisms that are cognitively robust in the sense that they are treated as a real observer despite of obvious evidence to the contrary. However, this finding is perhaps not so surprising, given that building up a reputation requires much time and energy and losing a good reputation can be quick and might have strong and lasting negative effects. Hence, the mere risk of losing a good reputation may have selected for high responsiveness towards critical cues (such as eyes watching) and corresponding changes in behaviour (i.e. enhanced cooperativeness of individuals that are watched) [23], [24], [27], [28], [30]. This is especially so under non-anonymous conditions in which individuals can monitor each other's behaviours most of the time, a situation which appears to have prevailed throughout large parts of human evolutionary history in small-scale societies.

Based on this reasoning, we suggest that the most parsimonious explanation for our results is that images of eyes triggered a reputational response and that the personal benefits individuals derived from maintaining a good reputations produced investments in the public good [31], [32]. This hypothesis is corroborated by several findings. Individuals invested more time in handling garbage in the presence of eyes compared to in the control situation. The reason appears to be that individuals handled more garbage items in the presence of eyes. We can exclude the possible alternative explanation that the images of eyes were more effective at attracting people's attention towards the symbolic sign compared to flowers, as people noticed the sign requesting garbage removal more often in the presence of flowers compared to in the presence of eyes.

A second potential explanation for our findings is that images of eyes have been perceived as a direct threat of punishment (instead of the more indirect threat of obtaining a bad reputation). Punishment has been suggested to enhance cooperation across taxa [33], [34] and a threat of punishment might have been the reason for why people invested more in the presence of cues of eyes. However, we are not aware of any study showing that images of eyes alone can indeed elicit a threat response. As yet it remains unclear whether this factor might have contributed in explaining our results.

### (b) Why did images of eyes only affect the behaviour of persons who engaged in garbage clearing?

About one third of the subjects cooperated by disposing garbage. This proportion seems relatively high, given that individuals did not obtain any material benefits from their contribution. Overall (including all individuals that were present at the bus stop) people were not more likely to engage in garbage clearing in the presence of images of eyes. Hence, it appears that cues of being watched were not sufficient in our setting to trigger a behavioural response. However, this interpretation should be treated with caution. Three reasons might have contributed to the lack of a treatment effect on whether or not people engaged in garbage clearing.

First, an implicit rule might have been acting only for people who wished to sit down. This is the rule: “If you produce garbage, then you must dispose of it properly.” As we placed the garbage ourselves at the bus stop, this rule likely did not affect people who remained in some distance from the bus stop bench. However for people who wished to sit down this rule likely became relevant. These people were in a way “forced” to “take possession” of the garbage, as a potential passer-by could assume that the passenger had generated the garbage and was littering. The finding that the motivation to sit down appeared to trigger removal of garbage corroborates this, as persons who sat down tended to be more likely to engage in garbage removal. However, if individuals only wanted to sit down, they could have done so by simply pushing aside a garbage item without even touching it with the hands (personal observation). Moreover, in order to sit down, it would have been sufficient to remove only one of the three items. Instead, half of the persons removed and deposited more than one garbage item into the bins. Hence, individuals invested more than necessary to sit down and this amount of surplus investment appears to have been modulated by the presence of eyes.

Secondly, not all people who were present at the bus stop might have been affected by the treatment. In contrast to the situation in a University kitchen, in which individuals prepared their drink in front of the experimental cues of being watched [27], people in our study, particularly when they did not approach the bench in order to sit down and therefore remained in some distance to the treatment, might not have been affected by the treatment. Including these cero values therefore potentially precluded finding any effect of the treatment.

Third, handling of foreign garbage creates a high threshold for actually engaging in behaviour as many people might sicken at the idea to handle potentially dirty foreign rubbish. The threshold involved in engaging in removal of foreign garbage (e.g., potential risks of infection) may be too high to be surpassed by subtle cues of being watched.

In our field study we encountered several additional sources of variation compared to other studies, partly resulting from our attempt to enhance realism. These may have increased the variance in our data and thereby might have decreased the size of the effect. By placing a sign to dispose garbage we may have raised the attention to an injunctive norm (disapproval of littering). Studies suggest that injunctive norms coupled with a conflicting descriptive norm (in our case the presence of litter indicating that people litter at this bus stop) might reduce the likelihood to engage in cooperative behaviour (here: antilittering) [35]. Furthermore, in our study, individuals were unfamiliar to each other; they were tested at pubic locations and were not tested more than once. Finally, we tested individuals at 14 different bus stops with potential local differences in the type of persons (the different locations in Geneva are inhabited by persons from different social and cultural groups) and other environmental variables.

### (c) Why is there mixed evidence concerning the effects of image of eyes?

Overall, a number of studies (including this study) found effects of cues of being watched on behaviour [23], [24], [27], [28] while others did not [36] or only when the audience was familiar [37]. In one study, the mere presence of observers had no effect on the tendency of proposers to cooperate in an ultimatum game, but proposers responded to the degree of anonymity and the presence or absence of familiar individuals [37]. Cues of eyes did also not affect the behaviour of players in a trust game [36]. A potential reason for this lack of effect might be that both these studies were framed in a game situation with two interacting parties. Consequently, people might have been focussing on the interaction (the action of the partner), which could have distracted them from the subtle cues of being watched by a third party. In accordance with this interpretation, most of the studies that found effects of cues of being watched took place in a public good situation in which individuals invest in a common pool [24], [27], [28]. Interaction effects with other participants in such a game appear to be limited. Two studies found effects in a dictator game [23], [25]. However, this “game” does not reflect a full-fledged interaction as only the proposer makes a decision about how to split a certain amount of money while the receiver passively accepts the money (which is why the dictator game is not formally considered a game). Overall, the combined results of previous studies and this study suggest that the subtle effects of cues of being watched by a third party can be cancelled out when individuals are directly interacting, but that they can take effect when individuals are not distracted by an interaction with others.

### (d) Why is it critical to conduct experiments under real life conditions?

Many studies that investigated contributions to public goods have been conducted under highly controlled lab conditions that allow for explicit testing of particular parameters of interest [19], [38]. While the results of these studies provide important information about the mechanisms underlying behaviour, the applicability of the findings to real-world settings requires to be determined [24], [27], [28], [39]. For instance, many lab studies are conducted using only a fraction of the population (University students in Western countries), which might bias the results and in the extreme may cause artefacts [40], [41]. Moreover, it is important to establish whether the behavioural responses persons show towards computer screens and under artificial lab conditions indeed reflect behaviour under more realistic settings. Studies on cooperation under controlled lab conditions typically operate with money gains and losses as a result of the interaction. Here we show that individuals increase investments based on reputational cues, even if no monetary rewards are involved. Other incentives than monetary gains or losses may often be involved in cooperative behaviour in real life. For instance, a study showed that individuals are more likely cease contributing to a public good if they perceive cues about free riding of others. This study showed that individuals were more likely to cheat by leaving kitchenware in the sink unwashed, when unwashed items were already present, compared to in the absence of unwashed items [12]. Other examples of non-monetary incentives enhancing cooperation include attractiveness to the opposite sex [42], or access to more cooperative partners [15].

### Conclusion

Theory and reality about human behaviour have repeatedly been shown to deviate considerably, one of the latest examples being the financial crisis [43]. To obtain a more accurate picture about the mechanisms underlying human cooperative behaviour, experiments under settings as realistic as possible are required [39]. Such experiments might lead to the conclusion that, instead of the complex cognitive processes that are often assumed to generally underlie human social behaviour, sometimes relatively simple and potentially unconscious mechanisms may be acting [44]. A better understanding of human cooperative behaviour in real life is of key interest both from theoretical and practical perspective. Several major problems humans face today such as climate change, financial crisis and over-exploitation of natural resources arise from the potential for a tragedy of the commons, i.e. a cooperation break down in a social dilemma [8], [45]. Field studies investing why individuals invest in a common good at a small scale are crucial to understand cooperation also in higher levels of human social organisation, i.e. organisations and nations [46].
